# Supplementary material for: Limited Effects of Class II Transactivator-Based Immunotherapy in Murine and Human Glioblastoma
Source: Cancers (Basel). 2023 Dec 30;16(1):193. doi: 10.3390/cancers16010193 (PMC10778432; doi:10.3390/cancers16010193)

SUPPLEMENTARY FIGURE S1. Western blot results showing CIITA expression in -WT versus -CIITA (a) GL261 murine cells and (c) U87, GM2, GM3, PGB13 and PGB-14 human (primary) glioblastoma cells. (b) FACS data indicating high IA/IE (MHC-II antibody) after transfection with the CIITA plasmid versus low IA/IE expression in wildtype cells.

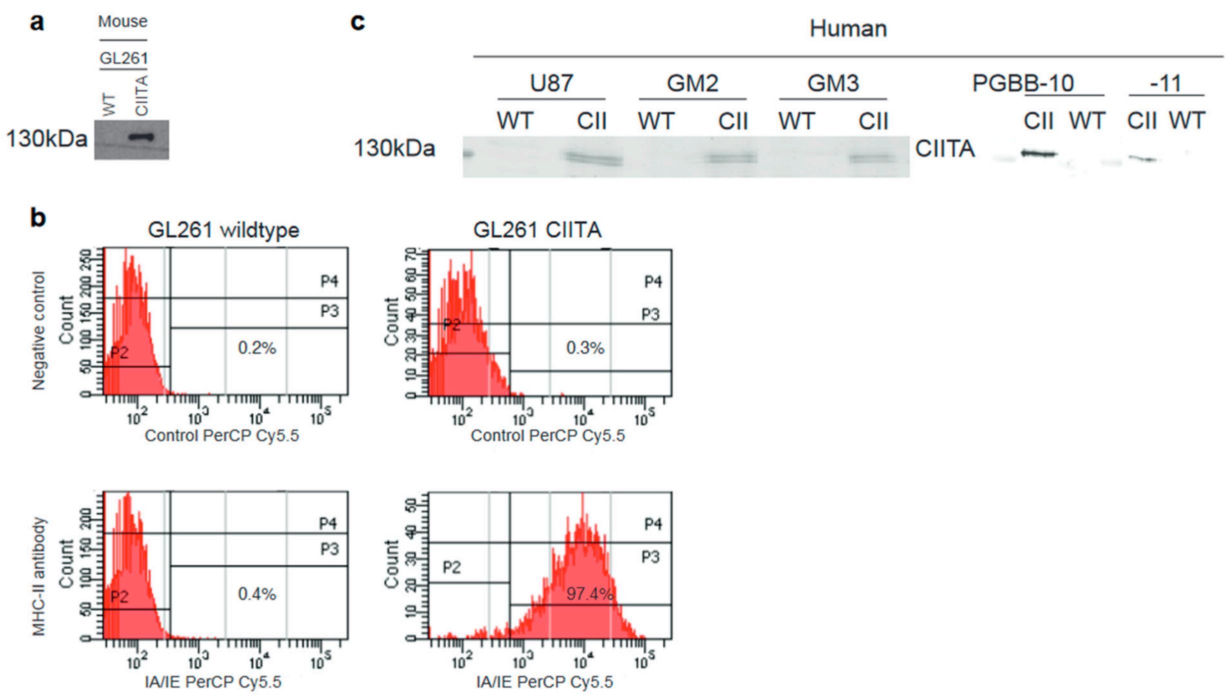

SUPPLEMENTARY TABLE S1. Histopathological diagnosis of glioma used for isolation of tumor-associated myeloid cells, tumor-infiltrating lymphocytes and NK-cells in co-culture experiments.

Patiënt	Histopathology	Experiment
PGB1	Glioblastoma WHO grade IV	TIL experiment
PGB2	Glioblastoma WHO grade IV	TIL experiment
PGB3	Glioblastoma WHO grade IV	TAM experiment
PGB4	Glioblastoma WHO grade IV	TAM experiment
PGB5	Glioblastoma WHO grade IV	TAM experiment
PGB6	Glioblastoma WHO grade IV	TAM experiment
PGB7	Glioblastoma WHO grade IV	TAM experiment
PGB8	Glioblastoma WHO grade IV	TAM experiment
PGB9	Diffuse astrocytoma WHO II	TAM experiment
PGB10	Diffuse low grade glioma WHO II	TAM experiment
PGB11	Gemistocytair astrocytoom WHO II	TAM experiment
PGB12	Diffuse astrocytoma WHO II	TAM experiment
PGB13	Glioblastoma WHO grade IV	NK-cell experiment
PGB14	Anaplastic oligodendroglioma WHO grade III	NK-cell experiment
PGB15	Glioblastoma WHO grade IV	NK-cell experiment
PGB16	Glioblastoma WHO grade IV	NK-cell experiment
PGB17	Low grade glioma WHO grade II	NK-cell experiment

SUPPLEMENTARY FIGURE S2. (a) CIITA and HLA-DR expression in U87-WT and U87-CIITA cells after co-culture with TAM. (b) TAM relative gene expression after co-culture differentiating between glioblastoma-derived (GBM) versus low-grade glioma derived (LGG) TAM. (c) TAM gene expression Each bar represents the mean ± SD relative gene expression with separate data points.

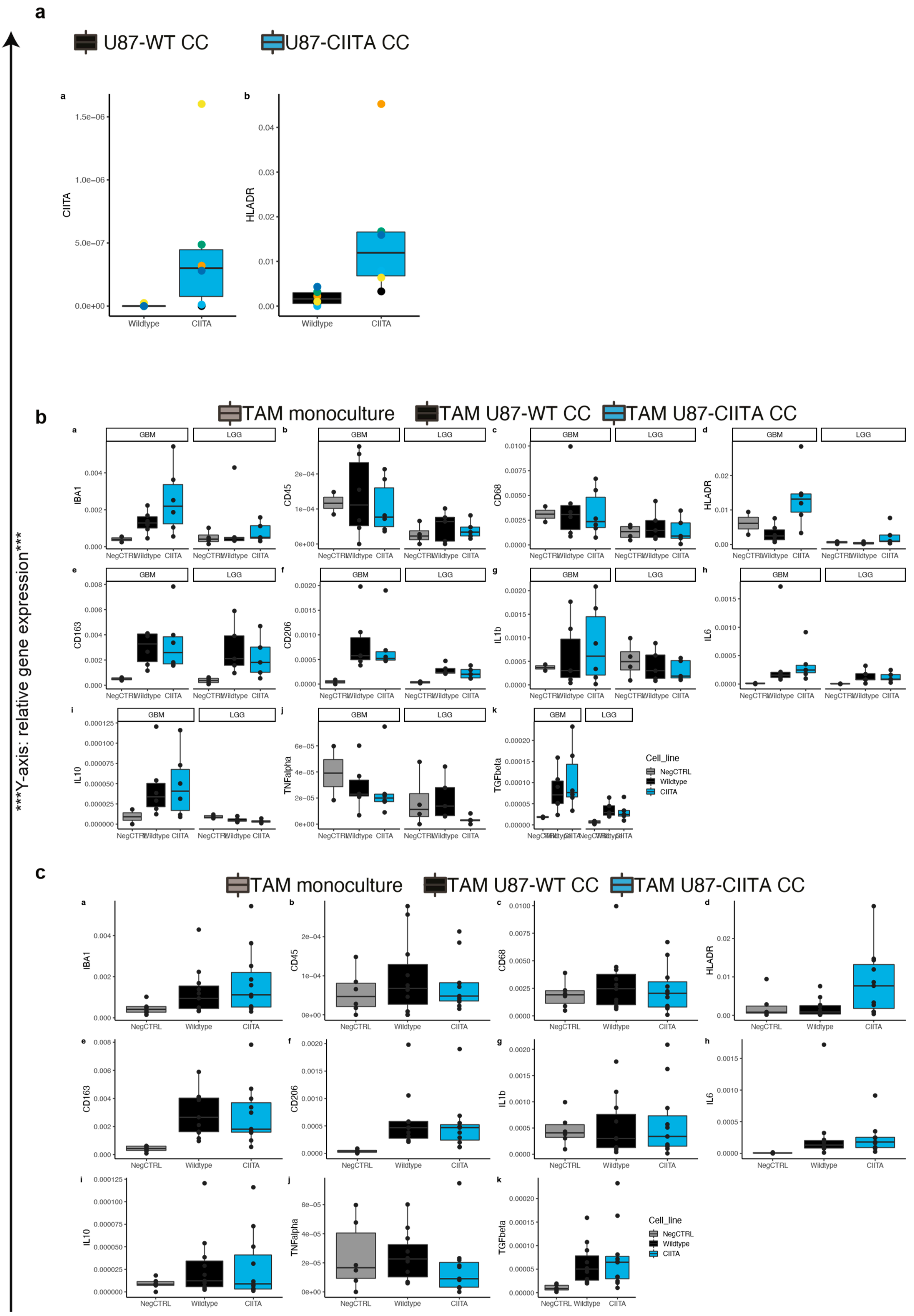

SUPPLEMENTARY FIGURE S3. (A) RT-qPCR analyzed relative gene expression of NK-cell related genes after co-culture with U87 (NK-cell markers (A) or tumor cell markers (B) or GM2 NK (C) versus tumor (D). Each bar represents the mean ± SD relative gene expression with separate data points.

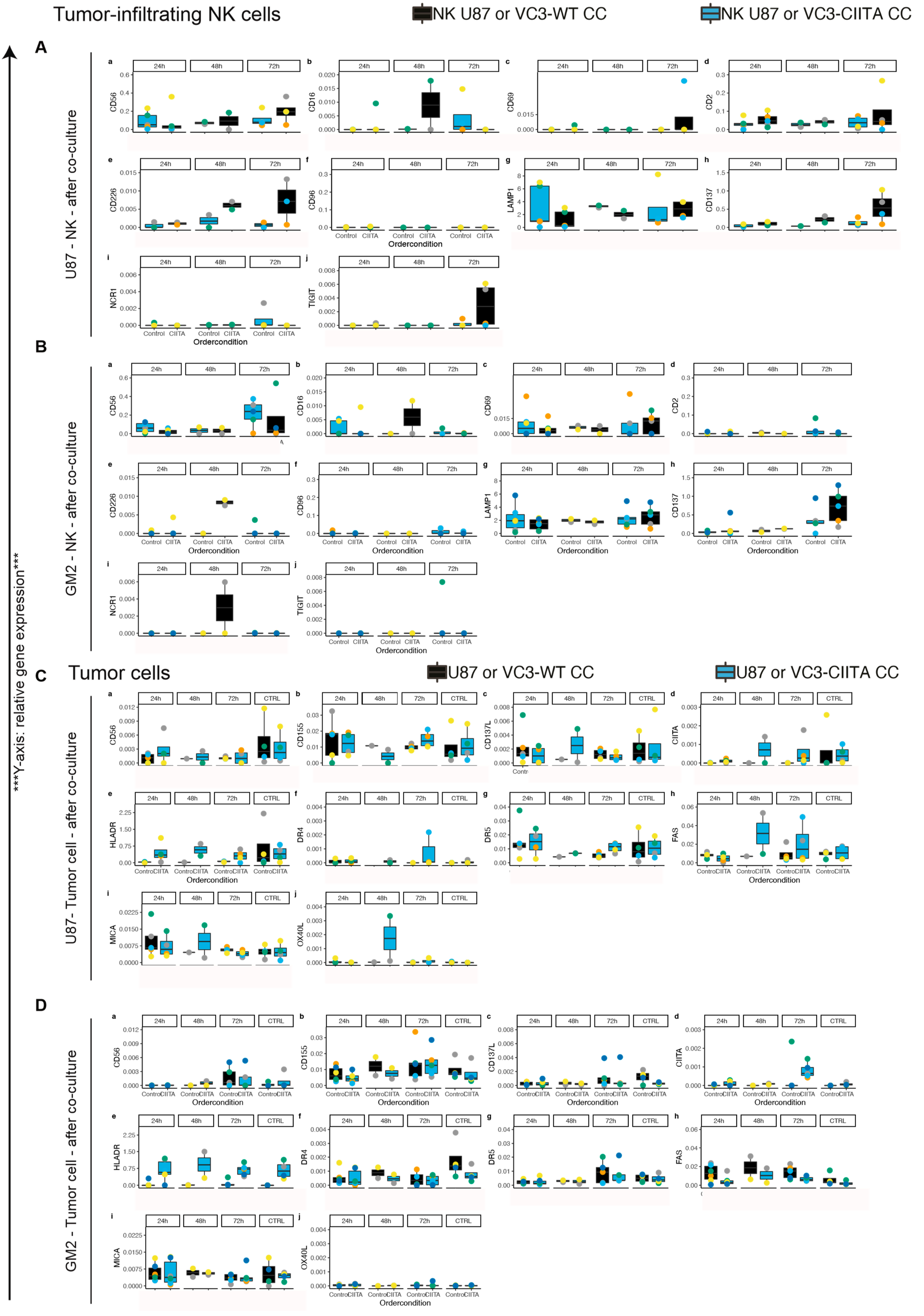

SUPPLEMENTARY FIGURE S4. (A) RT-qPCR analyzed relative gene expression of tumor-infiltrating lymphocytes. TIL-related genes (A) or tumor related genes \ (B) Each bar represents the mean ± SD relative gene expression with separate data points.

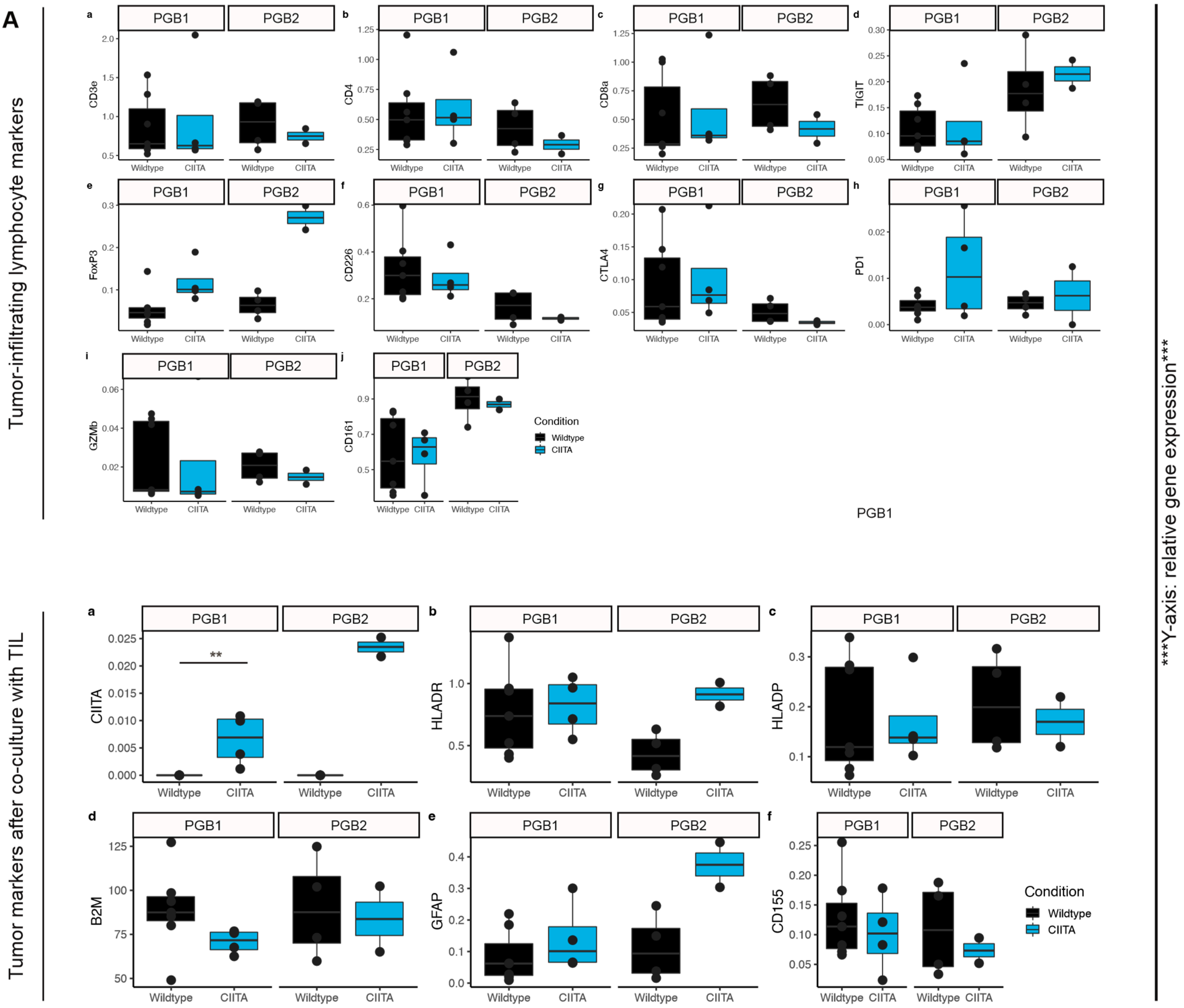

SUPPLEMENTARY TABLE S2. Primers pairs for tested genes.

Gene	Primers sequence		Gene	Primers sequence	
**General**	Forward	Reverse	**TIL**	Forward	Reverse
CIITA	GCTGGAGAAGAAGAGAT	CGCGATATTGGCATAAG	FoxP3	CTCAGCTTAGACTGCAGAGGAAC	TTATATGGGGTGATAGTCCAGCA
GFAP	TGGAGCTCAATGACCGCTTT	CCAGGAAGCGGACCTTCTC	CTLA4	GGCACCTCCAGTGGAAATCA	TCAGCACCACACTCACCATT
GAPDH	TGCACCACCAACTGCTTAGC	GGCATGGACTGTGGTCATGA	PD1	ACGAGGGACAATAGGAGCCAG	GCCCATTCCGCTAGGAAAGAC
RNA-polymerase II	GTTTTTCCGTGCTGTGTAGGG	TCATTGGTTGCTGCTTTGGA	CD161	TGCCATGGACCAACAAGCAA	CCAAGGTGAACCCTGACAGA
E2Ubi	CTGAAGAGAATCCACAAGGAATTGA	CTCCAACAGGACCTGCTGAAC	**NK-cells**		
TBP	CAGCTTCGGAGAGTTCTGGG	TATATTCGGCGTTTCGGGCA	CD56	ACTTATCGACCATGGAGCGC	AGCTCACAAGGTTCTGGCGT
**TAM**			CD155	AAAGAGGGACCTCCCAGTGA	GTGGATCCTGGGAAGAGCTG
IBA1	AGACGTTCAGCTACCCTGACTT	GGCCTGTTGGCTTTTCCTTTTCTC	CD137L	GAGCTTTCGCCCGACGAT	GTCACTGTACCAGCTCAGGG
CD45	GCAGCTAGCAAGTGGTTTGTTC	AAACAGCATGCGTCCTTTCTC	DR4	GTCTGTTGTTGCATCGGCTC	TGTGAGCATTGTCCTCAGCC
CD68	CTTCTCTCATTCCCCTATGGACA	GAAGGACACATTGTACTCCACC	DR5	TCCTGAGATGTGCCGGAAGT	CCACTGTGCTTTGTACCTGATTC
HLA-DR	CCCAGGGAAGACCACCTTT	CACCCTGCAGTCGTAAACGT	FAS	GAACACTGTGACCCTTGCAC	AAGACAAAGCCACCCCAAGT
CD163	TTTGTCAACTTGAGTCCCTTCAC	TCCCGCTACACTTGTTTTCAC	MICA	GCTGGCATCTTCCCTTTTGC	CTGCACAGATCCATCCCAGG
CD206	TGCAGAAGCAAACCAAACCTGTAA	CAGGCCTTAAGCCAACGAAACT	OX40L	TCTGCCTGCACTTCTCTGC	CCTCCTTTTGGGAAGTGAGGA
IL-1b	TTTGAGTCTGCCCAGTTCCC	TCAGTTATATCCTGGCCGCC	CD16	GACAGCGGCTCCTACTTCT	ATGGTTGACACTGCCAAACCT
IL-6	TGCAATAACCACCCCTGACC	TGCGCAGAATGAGATGAGTTG	CD69	TGCATCCGGAGAGTGGACAA	CACACAGGACAGGAACTTGGA
IL-10	GGCGCTGTCATCGATTTCTTC	CACTCATGGCTTTGTAGATGCC	CD2	GGCAGCCTCTTGATGGTCTT	CTGTGGGCTCTTGTCTCCAG
TNF-alpha	TGGAGAAGGGTGACCGACTC	TCACAGGGCAATGATCCCAA	CD96	GACCTCCAGTGGGACAGATAC	CATGAAGTGTTGAGCCTGCAC
TGF-beta	CAATTCCTGGCGATACCTCAG	GCACAACTCCGGTGACATCAA	LAMP1	GTAACGCTCCATGATGCCAC	CTGTCTTGTTCACAGCGTGTC
Beta-2-microglobulin	GTGCTCGCGCTACTCTCTCT	TCAATGTCGGATGGATGAAA	CD137	ACGCTCCGTTTCTCTGTTGT	GAAATCGGCAGCTACAGCCA
**TIL**			NCR1	CATCTATCGGGTTGGGGAGC	CGAGATCACTTCGGGTCCAG
CD3e	TATCAGTTGGCGTTTGGGGG	TCCAGGATACTGAGGGCATGT	TIGIT	TGGGACGTACACTGGGAGAA	TGGCTCCAAGCAATGGAATCT
CD4	TGCTGAGTGACTCGGGACAG	CCAGCACAATCAGGGCCATTG	CD226	TTAGAGCGAGCAGCACTCAC	TGCCAAAGCACCTCTTCACA
CD8a	ACTTGTGGGGTCCTTCTCCT	GTCTCCCGATTTGACCACAG	Granzyme B	TGGGGGACCCAGAGATTAAAA	TTTCGTCCATAGGAGACAATGC

SUPPLEMENTARY FIGURE S5. (a) RNA sequencing data showing HLA-I and -II expression in human glioblastoma celll ines. (b) Survival of patients with high (>0%) and no (0%) CIITA expression. (c) Survival curve of patients with high (>0%) or no (0%) HLA-DRa expression. (d) Survivval curve from TGCA database patients with with highest 90 and lowest 10th percentile and (e) highest 75 and lowest 25th percentile. (e) Caspase 3/7 apoptose assay in NK cell co-culture experiments. (f) Percentage of cells in apoptosis per time point, GM2-WT versus -CIITA.

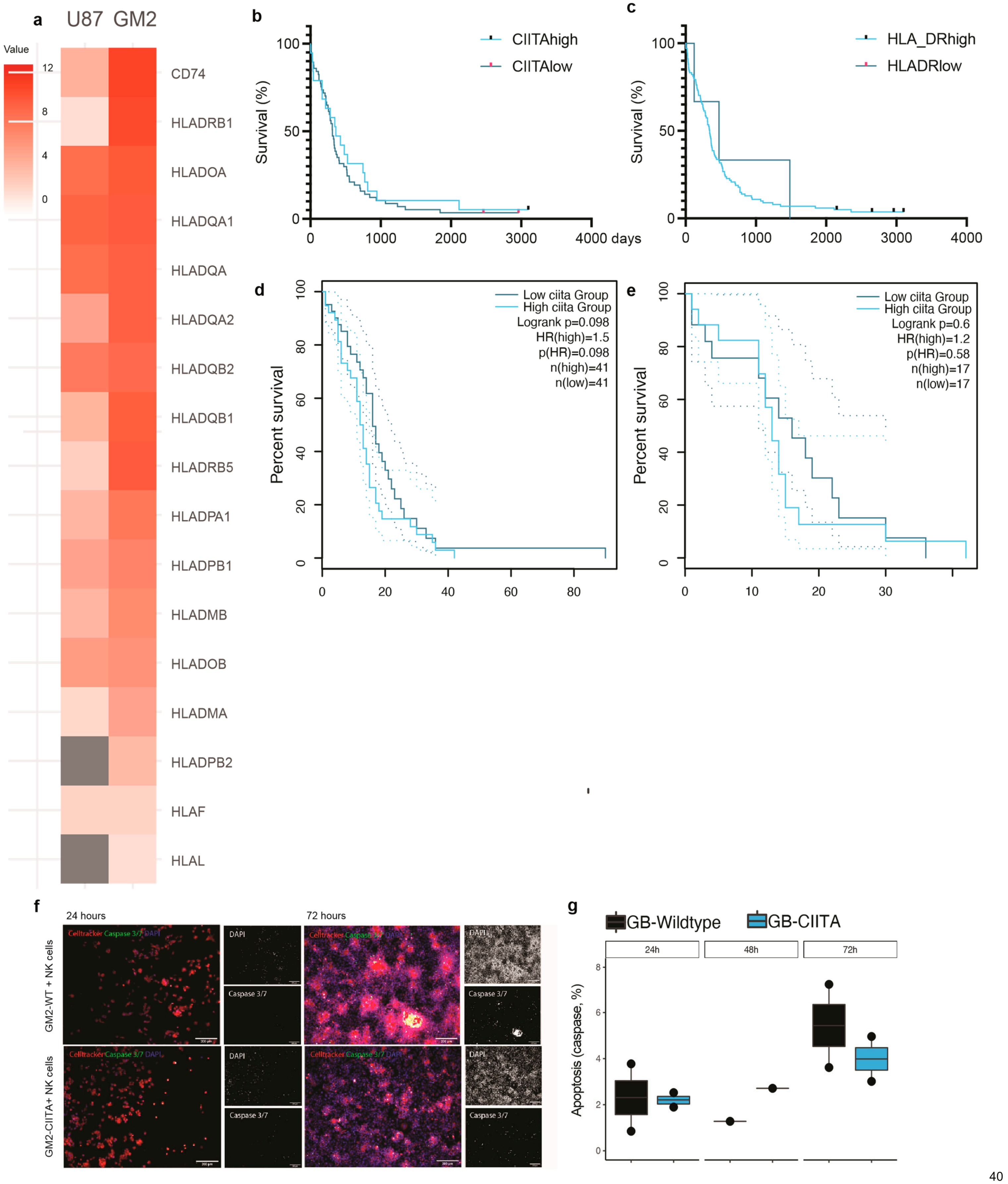

Supplement: Supplementary file 1 [file cancers-16-00193-s001.zip › cancers-2784692-supplementary.pdf]
